# Brain Morphometry Fingerprints are Associated with Gait Initiation Deficits in Parkinson’s Disease and Freezing of Gait

**DOI:** 10.1111/ejn.70647

**Published:** 2026-08-04

**Authors:** Luana dos Santos de Oliveira, Mariana Penteado Nucci, Carla Silva‐Batista, Caroline Ribeiro de Souza, Andrea Cristina de Lima‐Pardini, Fabio Augusto Barbieri, Daniel Boari Coelho

**Affiliations:** ^1^ Center for Mathematics, Computation and Cognition Federal University of ABC São Bernardo do Campo Brazil; ^2^ Hospital das Clínicas da Faculdade de Medicina da Universidade de São Paulo São Paulo Brazil; ^3^ Department of Neurology Oregon Health and Science University Portland Oregon USA; ^4^ Biomedical Engineering Federal University of ABC São Bernardo do Campo SP Brazil; ^5^ São Paulo State University (UNESP) School of Sciences, Department of Physical Education, Human Movement Research Laboratory (MOVI‐LAB) Bauru SP Brazil

**Keywords:** cortical thickness, movement disorders, postural control, supplementary motor area

## Abstract

Freezing of gait (FoG) is frequently triggered during gait initiation, a transition that depends on anticipatory postural adjustments (APAs). However, the neuroanatomical substrates linking brain morphometry to APA control during step initiation in FoG remain unclear. We investigated whether subcortical volumes and cortical thickness in motor–cognitive regions relate to APA features and clinical severity. Forty‐four right‐handed individuals with midstage PD (26 PD + FoG; 18 PD − FoG) performed step initiation. FreeSurfer‐derived regional volumes and cortical thickness were extracted. Associations between morphometry and APA metrics were tested across all participants and within groups. Group differences were tested using the Mann–Whitney *U* tests, and partial Spearman correlations examined morphometry–clinical and morphometry–APA associations, controlling for UPDRS‐III and daily levodopa dose. Compared with PD without FoG, the PD + FoG group showed higher UPDRS‐III scores and levodopa doses, lower APA mediolateral amplitudes, and larger amygdala volumes. Across all participants, longer APA duration was associated with smaller putamen, thalamus, and dorsolateral prefrontal cortex volumes, whereas greater APA mediolateral amplitude was associated with lower frontopolar cortical thickness. In PD + FoG, freezing severity and APA features were associated with distinct morphometric patterns across the basal ganglia, thalamus, prefrontal, cingulate, SMA, and insular regions. These findings suggest that gait initiation impairment in PD reflects distributed morphometric alterations rather than a single regional substrate. APA measures may provide mechanistically relevant biomechanical markers for identifying neural systems involved in FoG.

## Graphical abstract

The figure shows the location of the selected regions in sagittal, coronal, and axial views of a structural brain image. PD: putamen (blue), thalamus (green), DLPFC (red), frontopolar (yellow). PD + FoG: pallidum (pink), nucleus accumbens (blue/pink), frontopolar (yellow), thalamic (green), DLPFC (red), anterior midcingulate volumes (blue), and SMA (blue). PD‐FoG: pallidum (pink), nucleus accumbens (blue/pink), frontopolar (yellow), anterior midcingulate volumes (blue), and amygdala (red). APA, anticipatory postural adjustment; a.u., arbitrary units; DLPFC, dorsolateral prefrontal cortex; FoG: freezing of gait; ML, mediolateral; SMA, supplementary motor area.

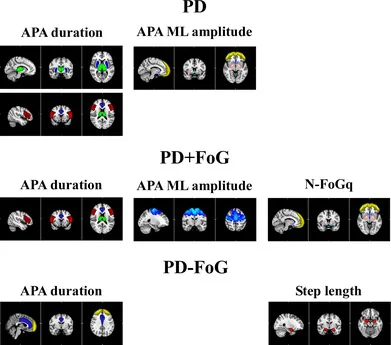

**Abbreviations**
APA: Anticipatory postural adjustment
a.u.: Arbitrary units
CoP: Center of pressure
DLPFC: Dorsolateral prefrontal cortex
FA: Flip angle
FoG: Freezing of gait
H&Y: Hoehn and Yahr
M1: Primary motor cortex
ML: Mediolateral
MoCA: Montreal Cognitive Assessment
MRI: Magnetic resonance imaging
N‐FoGq: New Freezing of Gait Questionnaire
PD: Parkinson's disease
PD + FoG: Parkinson's disease with freezing of gait
PD − FoG: Parkinson's disease without freezing of gait
ROI: Region of interest
SMA: Supplementary motor area
STN: Subthalamic nucleus
UPDRS‐III: Unified Parkinson's Disease Rating Scale, Part III

## 1 Introduction

Freezing of gait (FoG) is a disabling episodic gait disturbance in Parkinson's disease (PD), commonly triggered during gait initiation, a transition that requires rapid reconfiguration of the locomotor program (Gilat et al. 2026). Step initiation depends on anticipatory postural adjustments (APAs), which shift the center of pressure toward the stepping limb and help unload the swing leg (Delval et al. 2014; De Oliveira, Silva‐Batista, et al. 2025). Individuals with PD often show impaired gait initiation, including prolonged APAs and reduced step amplitude, and these deficits are typically more pronounced in those with FoG, particularly in the spatial scaling of mediolateral and anteroposterior APAs (De Oliveira, De Oliveira, et al. 2025; De Oliveira, Silva‐Batista, et al. 2025). Dysfunction within cortico–basal ganglia–SMA and brainstem locomotor networks may disrupt the internal cueing, preparation, and scaling of APAs, thereby contributing to impaired step release and FoG during gait initiation (Bardakan et al. 2022).

Neuroimaging studies suggest that FoG does not arise from a single lesion site but from distributed cortico–subcortical dysfunction involving motor automaticity, cognitive control, limbic function, and visuospatial processing (Herman et al. 2014; Vercruysse et al. 2015; Wang et al. 2016; Bharti et al. 2019; D'Cruz et al. 2021). Structural studies have implicated striatal and thalamic regions, with prior work linking freezing severity to putamen volume and reporting thalamic alterations in PD + FoG (Sunwoo et al. 2013; Rosenberg‐Katz et al. 2016). Multimodal studies further support this network perspective: Canu et al. (2015) reported white matter and resting‐state connectivity abnormalities across motor, prefrontal, parietal, default‐mode, and visual associative networks in PD + FoG, while Ragothaman et al. (2022) showed that brain volumes relate to objective balance and gait measures, including step initiation. More directly related to APAs, de Lima‐Pardini et al. (2020) reported altered SMA and anterior insula involvement during APA‐related movement in PD + FoG, supporting a role for motor‐preparatory and salience networks in the impairment of anticipatory control.

At the cortical level, gait initiation implicates a motor–cognitive network including the SMA, dorsolateral prefrontal cortex (DLPFC), frontopolar cortex, primary motor cortex (M1), and insula. These regions support internally generated movement, executive control, motor execution, and salience/interoceptive integration. Although alterations in cortical thickness in the SMA/cingulate and DLPFC have been reported in PD + FoG, findings remain heterogeneous (Li et al. 2020). More broadly, cortical morphometry has been linked to clinical manifestations of PD, while recent work suggests that gait performance may relate to specific structural or microstructural markers rather than cortical thickness alone (Deng et al. 2016; De Oliveira, De Oliveira, et al. 2025; Fukuchi et al. 2025; Wróbel et al. 2025).

A key limitation of the existing literature is that neuroanatomical associations are typically tested against clinical FoG questionnaires, composite gait scores, or broad motor severity metrics, rather than against mechanistically interpretable, task‐evoked biomechanical markers of gait initiation. In particular, APAs provide a direct window into the feedforward postural control processes that must be engaged before the first step, yet the structural neuroanatomical substrates supporting interindividual differences in APA magnitude and timing, especially in PD + FoG, remain insufficiently characterized. This gap matters because structural correlates of FoG have been inconsistently localized across studies. Clarifying which regions relate to specific gait initiation control variables could refine mechanistic models and improve intervention targeting (Herman et al. 2014; Rubino et al. 2014).

Therefore, the present study investigated whether regional brain volumes and cortical thickness measures are associated with APA features during step initiation in individuals with midstage Parkinson's disease who have FoG (PD + FoG) and those without FoG (PD − FoG). Based on prior evidence indicating that FoG and gait initiation deficits in PD involve distributed cortico–subcortical networks supporting motor automaticity, postural preparation, executive control, and limbic/salience processing, we hypothesized that, across all participants, poorer APA organization, particularly longer APA duration, would be associated with reduced morphometry in striato–thalamo–limbic and prefrontal regions, including the caudate, putamen, thalamus, amygdala, DLPFC, and frontopolar cortex. For the PD + FoG group, we further hypothesized that these associations would be stronger and more widespread, reflecting the greater dependence of freezing‐related gait initiation on impaired basal ganglia–thalamic gating, cognitive control, and internally generated movement preparation. In particular, we expected lower striatal and thalamic volumes to be associated with prolonged APA timing and reduced SMA/mesial frontal morphometry to be associated with less effective APA control, given the established role of the SMA and cingulate regions in self‐initiated movement, postural preparation, and FoG‐related cortical alterations. For the PD − FoG group, we hypothesized that brain–APA associations would be weaker and less regionally extensive because the literature more consistently links pronounced gait initiation impairment and freezing severity to distributed cortico–basal ganglia–thalamic dysfunction in PD + FoG. Finally, given the heterogeneous evidence regarding cortical thickness as a gait‐relevant marker in PD, we considered associations with cortical thickness, particularly in SMA, DLPFC, frontopolar cortex, M1, and insula, as biologically plausible but more exploratory than volumetric associations.

## 2 Methods

### 2.1 Participants

Forty‐four right‐handed (26 with PD + FoG), midstage patients with PD, diagnosed according to the UK Brain Bank criteria (Hughes et al. 1992), as determined by the Edinburgh Handedness Inventory, participated in this study. Inclusion criteria were as follows: (1) a Hoehn & Yahr (H&Y) score ≤ 4; (2) availability to engage in the task training, brain imaging, and neuropsychological assessments; (3) capacity to walk independently, without assistance devices; (4) ability to understand instructions to perform the experimental motor tasks; (5) absence of neurological diseases other than PD; and (6) absence of musculoskeletal impairments possibly affecting performance of the experimental tasks. The exclusion criteria were as follows: (1) difficulty performing the experimental motor tasks, (2) dropout from any assessment, (3) any contraindication to undergoing an MRI exam, and (4) poor quality of the brain volumes acquired during the MRI: head motion above 1 mm (Seto et al. 2001). Individuals were classified as having FoG if they answered the first question of the New FoG Questionnaire (N‐FoGq) (Nieuwboer et al. 2009) affirmatively, and FoG status was additionally confirmed by a physiotherapist specialized in movement disorders during a provocative walking circuit designed to elicit FoG episodes. The circuit included walking through doorways, zig‐zag walking, and turning maneuvers, which are common situations associated with FoG occurrence. The physiotherapist directly observed participants during the circuit and confirmed the presence of FoG episodes. All the FoG individuals scored 3 or 4 in the N‐FoGq question: “How frequently do you experience episodes of freezing when initiating the first step?” indicating moderate or severe FoG during step initiation. All evaluations were performed during the ON state of dopaminergic medications, within 2 h of the first morning levodopa dose. All participants provided informed consent, and the experimental procedures were approved by the Institutional Review Board of the University of São Paulo.

### 2.2 Experimental Design and Procedures

A skilled, experienced examiner conducted PD‐specific clinical and cognitive evaluations of the subjects. The H&Y staging, Unified Parkinson's Disease Rating Scale motor score (UPDRS‐III), and Montreal Cognitive Assessment (MoCA) were used to assess clinical characteristics. Participants were to take a step by moving their right foot. Both feet were positioned on a force platform (AMTI OR6–6, 200 Hz). A reflective marker was placed on the right malleolus to record foot movement using a Vicon system (200 Hz). The participants performed 20 step initiation trials. Participants were instructed to stand feet apart in a comfortable position on the force plate, and the foot positions were then marked with tape. On each trial, the participant was instructed to take a step, starting with the right leg, at a self‐selected, comfortable speed after the beginning signal. After each trial, the participant was to return to the initial position marked on the force plate.

Fatigue and learning effects were minimized through prior familiarization, self‐selected comfortable stepping speed, and rest periods when needed; no participant reported fatigue during the assessment. APA assessment, clinical evaluations, and MRI acquisition were performed within a maximum interval of 1 week for each participant. Data were processed offline and calculated by an assessor blinded to participants' FoG classification.

### 2.3 Image Acquisition

MRI data were collected on a 3.0‐T Philips Achieva whole‐body system (Eindhoven, Netherlands) using an 8‐channel head coil. To limit involuntary head motion, two adhesive tape strips were applied (one over the frontal bone and another over the mandibular mental protuberance) and foam padding was placed laterally and superiorly around the head. Earplugs were provided to attenuate scanner noise during acquisition. In addition, Velcro straps were used to reduce trunk movement. Participants were instructed to remain still for the entire scanning session.

High‐resolution anatomical images were acquired in the sagittal plane with a three‐dimensional T1‐weighted sequence. This sequence was selected because it provides a strong contrast among gray matter, white matter, and cerebrospinal fluid (CSF), enabling accurate FreeSurfer‐based segmentation and parcellation, as well as high‐fidelity morphometric quantification of cortical and subcortical structures. Imaging was performed using a 3DT1‐weighted sequence turbo factor (TFE) with the following parameters: repetition time (TR) = 7000 ms, echo time (TE) = 3.2 ms, matrix = 256 × 256, slice thickness (SC) = 1 mm, field of view (FOV) = 240 × 240 mm^2^, flip angle (FA) = 8°, number of slices = 180, voxel size = 1 × 1 × 1 mm^3^, and acquisition time (TA) = 5 min 21 s.

Brain morphometry was conducted with FreeSurfer (version 7.1.0) following the standard processing workflow for cortical and subcortical segmentation using the RECON‐ALL pipeline (http://surfer.mnr.mgh.harvard.edu/) (Tanner et al. 2017). Subcortical volumes were extracted from the FreeSurfer automated segmentation (aseg) output. Cortical volumes and cortical thickness were extracted from the FreeSurfer cortical parcellation (aparc, Desikan–Killiany atlas). For bilateral regions, left and right hemisphere volumes were summed. Cortical thickness values were averaged across hemispheres for each ROI. The volumes of the following regions of interest were computed as combined (left + right) measures: caudate, putamen, pallidum, thalamus, nucleus accumbens, brainstem, amygdala, supplementary motor area (SMA), dorsolateral prefrontal cortex (DLPFC), anterior middle cingulate cortex, frontopolar cortex, primary motor cortex (M1), and insula. Cortical thickness was additionally quantified in the SMA, DLPFC, frontopolar, M1, and insula. The DLPFC was operationalized as the combined rostral and caudal middle frontal parcels, whereas the SMA was defined using the medial/superior frontal motor parcel available in the FreeSurfer cortical parcellation. M1 was defined as the precentral gyrus, frontopolar cortex as the frontal pole, and insula as the insula parcel. In an additional exploratory analysis, cerebellar cortex and cerebellar white matter volumes were also extracted from the FreeSurfer automated segmentation output. Left and right hemisphere volumes were summed, and cerebellar volumes were adjusted for total intracranial volume using the same residual normalization approach applied to the other regional volumetric measures.

Because the resulting maps are not constrained by the native voxel resolution, they can capture submillimeter group differences. All segmentation and parcellation outputs were visually reviewed and approved as part of quality‐control procedures in Freeview. FreeSurfer‐derived morphometric measures have demonstrated good test–retest reliability across scanner manufacturers and magnetic field strengths (Reuter et al. 2010). Finally, regional volumes were adjusted and normalized for total intracranial volume using the residual normalization approach to account for interindividual differences in head size, consistent with established practice and prior studies (Hansen et al. 2015).

### 2.4 Biomechanical Analysis

Data were visually inspected. The APA began with the initial lateral displacement of the center of pressure (CoP) toward the leading limb. The end of the APA, and therefore the start of the step, occurred when the free vertical moment changed direction (i.e., became negative) upon initiation with the right limb (Moineau et al. 2014). The following variables were analysed: (a) APA duration, the time between the beginning of the APA and the beginning of the step; (b) APA mediolateral (ML) amplitude, the maximum amplitude of the last peak of mediolateral displacement of the CoP before the start of the step; and (c) step length.

### 2.5 Statistical Analysis

Data were screened for completeness and visually inspected prior to inferential testing. Because several variables were not expected to meet the assumptions of normality, group comparisons between PD + FoG and PD − FoG were conducted using nonparametric tests. Specifically, continuous outcomes were compared using the Mann–Whitney *U* test, and effect sizes were reported. Partial Spearman's rank correlation coefficients were computed with UPDRS‐III and daily levodopa dose entered as covariates, both across all participants and separately within the PD + FoG and PD − FoG groups. These analyses tested associations between clinical freezing severity (N‐FoGq), APA‐derived biomechanical variables (APA duration, APA mediolateral amplitude, and step length), and FreeSurfer‐derived regional brain volumes and cortical thickness measures. For volume measures, residualized intracranial volume‐adjusted values were used as described in the morphometry procedures.

To summarize the distributed morphometric pattern associated with freezing severity, we additionally calculated an exploratory FoG Severity Morphometric Fingerprint Index. This index was based on the regions that showed significant associations with N‐FoGq scores in the PD + FoG group in the primary analyses: pallidum volume, nucleus accumbens volume, and frontopolar cortex volume. Each regional volume was standardized to *z*‐scores across the entire sample, and the index was calculated as the average of these three standardized values. Therefore, lower values indicate a smaller combined morphometric profile across regions associated with greater freezing severity. The index was compared between PD + FoG and PD − FoG participants using the Mann–Whitney U test.

All tests were two‐tailed, and the significance level was set at α = 0.05. All statistical analyses were performed in JASP (Version 0.95).

## 3 Results

PD + FoG presents higher values in equivalent L‐DOPA dose and UPDRS‐III compared to PwPD‐FoG (Table 1).

**TABLE 1:** Means (standard deviations) of the clinical characteristics of the participants separately by group.

	PD + FoG (*n* = 26)	PD‐FoG (*n* = 18)	*p*
Age (years)	63.4 ± 6.6	67.3 ± 6.2	0.091
Male/female (*n*)	16/10	12/6	—
Disease duration (years)	9.3 ± 4.6	8.6 ± 4.8	0.608
Hoehn and Yahr			
H&Y 2 (*n*)	2	2	
H&Y 3 (*n*)	22	15	
H&Y 4 (*n*)	2	1	
L‐DOPA equivalent units (mg•day^−1^)	794.3 ± 459.2	414.4 ± 216.2	0.001*
MoCA (score)	21.1 ± 1.7	21.1 ± 1.8	0.970
N‐FoGq (score)	19.8 ± 6.1	0	—
UPDRS‐III (score)	38.5 ± 9.8	30.1 ± 8.8	0.006*

Abbreviations: MoCA, Montreal Cognitive Assessment; N‐FoGq, New Freezing of Gait Questionnaire; UPDRS‐III: motor score Unified Parkinson's disease rating.

* indicates a significant difference.

PD + FoG presents lower values in APA ML amplitude and higher amygdala and cerebellum white matter volume compared to PD‐FoG (Table 2).

**TABLE 2:** Means (standard deviations) of biomechanical and brain characteristics of participants by group.

Characteristics	PD + FoG (*n* = 26)	PD‐FoG (*n* = 18)	*p*
Biomechanical variables			
APA duration (ms)	530.04 ± 165.08	502.94 ± 94.23	0.948
APA ML amplitude (mm)	41.73 ± 9.11	52.55 ± 13.44	0.013*
Step length (mm)	379.82 ± 63.36	428.89 ± 82.46	0.056
Brain volumes (×10^−3^ norm)			
Caudate	4.59 ± 0.57	4.87 ± 0.46	0.131
Putamen	7.06 ± 0.96	6.81 ± 0.89	0.375
Pallidum	2.03 ± 0.25	2.01 ± 0.21	0.897
Thalamus	9.01 ± 0.76	8.70 ± 0.82	0.281
Nucleus accumbens	0.81 ± 0.16	0.68 ± 0.12	0.060
Brain stem	15.03 ± 1.21	14.50 ± 1.31	0.281
Amygdala	2.34 ± 0.34	2.17 ± 0.23	0.030*
SMA	3.04 ± 0.40	3.01 ± 0.43	0.697
DLPFC	29.49 ± 2.66	29.34 ± 2.41	0.991
Frontopolar	4.53 ± 0.40	4.45 ± 0.56	0.859
M1	16.53 ± 1.39	16.62 ± 1.63	0.972
Insula	10.70 ± 1.13	10.79 ± 1.09	0.859
Cerebellum white matter	18.65 ± 2.05	20.44 ± 2.34	0.013*
Cerebellum cortex	771.42 ± 412.08	834.70 ± 410.89	0.619
Cortical thickness (mm)			
SMA	2.17 ± 0.14	2.13 ± 0.18	0.337
DLPFC	2.50 ± 0.13	2.56 ± 0.14	0.172
Frontopolar	2.26 ± 0.14	2.19 ± 0.14	0.231
M1	2.16 ± 0.10	2.12 ± 0.15	0.187
Insula	2.86 ± 0.12	2.85 ± 0.12	1.000

Abbreviations: DLPFC, dorsolateral prefrontal cortex; M1, primary motor area; SMA, supplementary motor area.

* indicates a significant difference.

**FIGURE 1:**
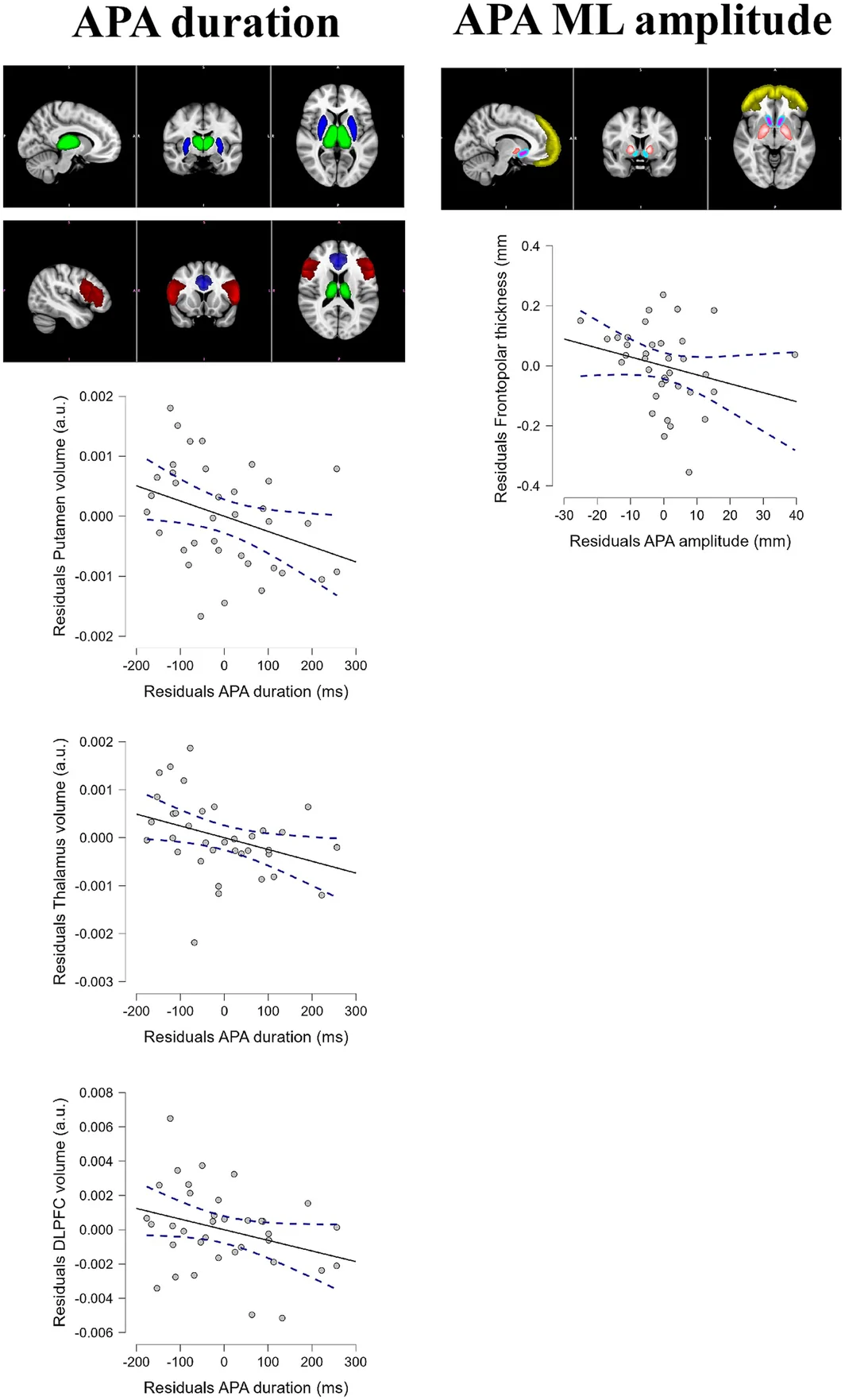
The figure shows the location of the selected regions in sagittal, coronal, and axial views of a structural brain image: putamen (blue), thalamus (green), DLPFC (red), frontopolar (yellow). Scatterplots show significant associations between residualized clinical/biomechanical variables and FreeSurfer‐derived regional volumes or cortical thickness after controlling for UPDRS‐III and daily levodopa equivalent dose. Associations are shown for the whole PD sample. Solid lines represent fitted linear trends, and dashed lines indicate 95% confidence intervals. APA: anticipatory postural adjustment; ML: mediolateral; DLPFC: dorsolateral prefrontal cortex; a.u.: arbitrary units.

Across all participants (Figure 1), longer APA duration was associated with smaller putamen (rho = −0.40, *p* = 0.019), thalamus (rho = −0.46, *p* = 0.007), and DLPFC volumes (rho = −0.38, *p* = 0.026). Larger APA ML amplitude was associated with smaller frontopolar thickness (rho = −0.39, *p* = 0.025). No significant associations were observed between APA duration and step length with cortical thickness. APA ML amplitude and step length were not significantly correlated with brain volumes. The FoG Severity Morphometric Fingerprint Index did not differ significantly between PD + FoG and PD − FoG participants (*p* = 0.135).

**FIGURE 2:**
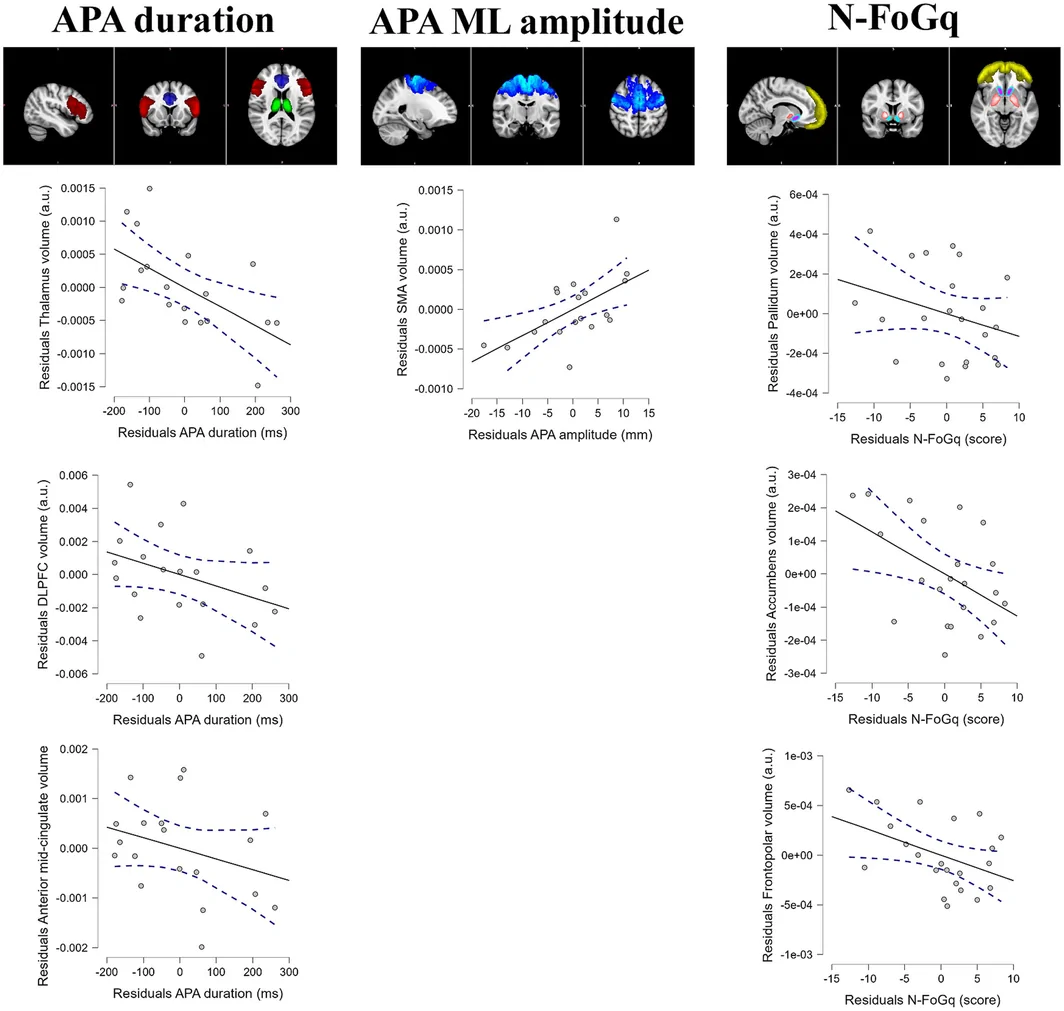
The figure shows the location of the selected regions in sagittal, coronal, and axial views of a structural brain image: pallidum (pink), nucleus accumbens (blue/pink), frontopolar (yellow), thalamic (green), DLPFC (red), anterior midcingulate volumes (blue), and SMA (blue). Scatterplots show significant associations between residualized clinical/biomechanical variables and FreeSurfer‐derived regional volumes or cortical thickness after controlling for UPDRS‐III and daily levodopa equivalent dose. Associations are shown for the PD + FoG group. Solid lines represent fitted linear trends, and dashed lines indicate 95% confidence intervals. APA: anticipatory postural adjustment; ML: mediolateral; DLPFC: dorsolateral prefrontal cortex; SMA: supplementary motor area; FoG: freezing of gait; a.u.: arbitrary units.

In the PD + FoG group (Figure 2), freezing clinical severity and APA measures showed partially distinct patterns of morphometric associations. Higher N‐FoGq scores were associated with smaller volumes in the pallidum, nucleus accumbens, and frontopolar cortex (pallidum: rho = −0.41, *p* = 0.038; nucleus accumbens: rho = −0.40, *p* = 0.041; frontopolar: rho = −0.45, *p* = 0.021) but not with cortical thickness. Longer APA duration was associated with smaller thalamic, DLPFC, and anterior midcingulate volumes (thalamus: rho = −0.66, *p* = 0.004; DLPFC: rho = −0.51, *p* = 0.037; anterior midcingulate: rho = −0.56, *p* = 0.020), with no significant associations with cortical thickness. Greater APA ML amplitude was associated with larger SMA volume (rho = 0.50, *p* = 0.039). Step length was not associated with cortical thickness but was negatively associated with insula volume (rho = −0.50, *p* = 0.040).

**FIGURE 3:**
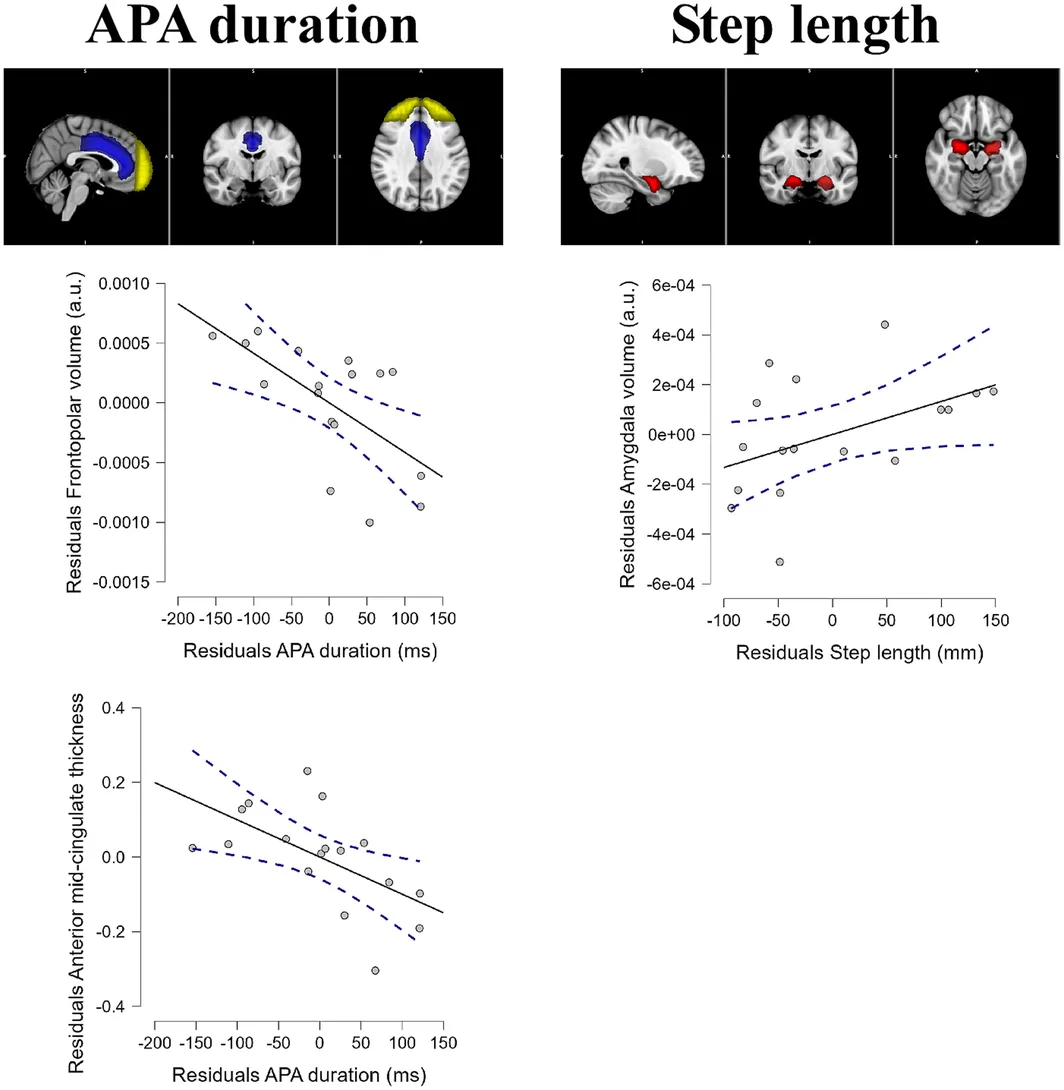
The figure shows the location of the selected regions in sagittal, coronal, and axial views of a structural brain image: pallidum (pink), nucleus accumbens (blue/pink), frontopolar (yellow), anterior midcingulate volumes (blue), and amygdala (red). Scatterplots show significant associations between residualized clinical/biomechanical variables and FreeSurfer‐derived regional volumes or cortical thickness after controlling for UPDRS‐III and daily levodopa equivalent dose. Associations are shown for the PD‐FoG group. Solid lines represent fitted linear trends, and dashed lines indicate 95% confidence intervals. APA: anticipatory postural adjustment; FoG: freezing of gait; a.u.: arbitrary units.

In the PD − FoG group (Figure 3), morphometric associations were more restricted and involved different regions. Longer APA duration was associated with smaller frontopolar volume (rho = −0.69, *p* = 0.004) and lower anterior midcingulate cortical thickness (rho = −0.57, *p* = 0.026). APA ML amplitude was not significantly correlated with brain volumes or cortical thickness. Step length was positively correlated with amygdala volume (rho = 0.53, *p* = 0.044) but not with cortical thickness.

The results of the other correlations are presented in the Supporting Information.

## 4 Discussion

The main finding of this study was that brain morphometry was associated with specific features of gait initiation in Parkinson's disease, with partially distinct patterns in individuals with and without FoG. Across all participants, longer APA duration was related to smaller putamen, thalamic, and DLPFC volumes, while greater APA mediolateral amplitude was associated with lower frontopolar cortical thickness. In the PD + FoG group, freezing severity was associated with smaller volumes in the pallidum, nucleus accumbens, and frontopolar cortex, whereas APA duration was associated with smaller volumes in the thalamus, DLPFC, and anterior midcingulate cortex. In this group, APA mediolateral amplitude also showed a positive association with SMA volume, and step length was negatively associated with insula volume. In contrast, the PD − FoG group showed a more restricted pattern, with longer APA duration associated with smaller frontopolar volume and lower anterior midcingulate thickness, while step length was positively associated with amygdala volume. Together, these findings suggest that APA timing, APA scaling, and step execution may relate to distinct morphometric substrates and that these associations vary with FoG status.

These findings fit well within a distributed‐systems framework of FoG. As emphasized by Weiss et al., FoG likely emerges from failure across a multilevel locomotor network, in which structural vulnerability, cognitive and limbic modulators, and dynamic network integration jointly determine whether gait initiation succeeds or breaks down (Weiss et al. 2020). Therefore, the present results should not be interpreted as identifying a single anatomical “center” for FoG. Instead, they suggest that impaired APA organization may be a behavioral expression of distributed network vulnerability, especially when postural preparation must be coordinated with motor release, cognitive control, and contextual evaluation.

The subcortical findings provide an important bridge between previous morphometric studies of FoG and the biomechanics of gait initiation. Across all participants, longer APA duration was associated with smaller putamen and thalamic volumes; in PD + FoG, it was also associated with a smaller thalamic volume. The putamen and thalamus are central nodes in basal ganglia–thalamo–cortical loops involved in movement preparation, internal cueing, and the release of voluntary motor programs. Smaller volumes in these regions may therefore contribute to a slower or less efficient feedforward organization of APAs. This interpretation is consistent with previous work linking freezing‐related phenotypes to striatal morphometry and reporting thalamic involvement in PD + FoG (Sunwoo et al. 2013; Herman et al. 2014; Rosenberg‐Katz et al. 2016).

The thalamic result also deserves a cautious interpretation. Although our study found that smaller thalamic volume was associated with longer APA duration, D'Cruz et al. (2021) reported that local thalamic enlargement predicted future FoG onset. These findings are not necessarily contradictory. D'Cruz et al. examined local thalamic morphology longitudinally as a predictor of conversion to FoG, whereas our study examined cross‐sectional regional volume in individuals already classified as PD + FoG or PD − FoG. Thus, thalamic involvement in FoG may follow a nonlinear trajectory, in which early local enlargement, compensatory remodeling, or altered thalamo–cortical coupling may precede later structural vulnerability. In our dataset, smaller thalamic volume appears to be more closely related to inefficient APA timing than to categorical FoG status alone.

The prefrontal and cingulate findings suggest that gait initiation in PD is strongly shaped by executive and action‐monitoring systems. Across all participants, longer APA duration was associated with smaller DLPFC volume, and in PD + FoG, this same association was accompanied by smaller anterior midcingulate volume. In PD − FoG, longer APA duration was associated with smaller frontopolar volume and lower anterior midcingulate thickness. These regions support executive control, goal maintenance, conflict monitoring, and the selection of appropriate actions. During gait initiation, especially in people with PD, stepping may require greater top–down control to compensate for impaired automatic basal ganglia gating. Reduced morphometry in the DLPFC, frontopolar, and anterior midcingulate regions may therefore manifest as delayed APA organization, reflecting greater difficulty with the first step of preparation and release. This interpretation is consistent with broader models of FoG as a disorder of distributed motor–cognitive control rather than purely motor failure (Canu et al. 2015; Sarasso et al. 2022).

The SMA finding in PD + FoG is particularly relevant from a mechanistic standpoint. Greater APA mediolateral amplitude was associated with larger SMA volume, suggesting that SMA integrity may support the spatial scaling of the lateral weight shift required for step initiation. This aligns with prior work showing that the SMA contributes to APA timing and self‐initiated movement and with recent neuroimaging evidence implicating SMA structure and microstructure in gait impairment and PD motor heterogeneity (Jacobs et al. 2009; Coelho et al. 2021; Fukuchi et al. 2025; Martin et al. 2025; Wróbel et al. 2025). In this sense, the SMA may represent a cortical node through which freezers preserve or compensate for the ability to scale APAs, even when broader basal ganglia–thalamo–prefrontal circuits are compromised.

The association between freezing severity and reduced volumes in the smaller pallidum, nucleus accumbens, and frontopolar cortex in PD + FoG further supports the idea that FoG involves more than the classical motor circuit. The pallidum is a major output node of basal ganglia circuitry, while the nucleus accumbens links motivational, limbic, and motor processes. Their association with N‐FoGq scores suggests that clinical freezing severity may reflect the combined vulnerability of motor gating and motivational/salience networks. The frontopolar cortex may add another layer, related to higher order control, prospective planning, and the ability to maintain goals during complex transitions such as gait initiation. This pattern is compatible with studies showing that FoG is associated with dysfunction across motor, cognitive, and limbic networks (Canu et al. 2015; Weiss et al. 2020).

The findings from the insula and amygdala should be interpreted with caution but are clinically interesting. In PD + FoG, step length was negatively associated with insula volume, whereas in PD − FoG, it was positively associated with amygdala volume. The insula is involved in salience detection, interoceptive awareness, and the integration of bodily state with action readiness. Its relationship with step length may indicate that step execution is influenced by the integration of internal bodily signals and contextual demands during movement preparation. The amygdala, although not classically considered part of the core gait initiation network, may influence stepping through emotional and contextual processing, including threat evaluation and fear of falling. Prior work has shown that amygdala–putamen and amygdala–frontoparietal connectivity are associated with FoG severity (de Lima‐Pardini et al. 2020). Thus, limbic structures may shape gait initiation indirectly by modulating cortico–basal ganglia circuits involved in APA timing, motor readiness, and step release.

Finally, cortical thickness showed fewer associations than regional volume measures. This does not necessarily mean that cortical morphology is irrelevant to gait initiation. Rather, average cortical thickness may be too coarse to capture the structural features most relevant to gait control. Prior work suggests that cortical morphology in PD may depend on the interplay between different surface‐based measures, such as cortical thickness and curvature, and that microstructural markers may be more sensitive to gait‐related impairment than thickness alone (Almgren et al. 2023; Wróbel et al. 2025). Therefore, the present findings support the use of multimodal structural approaches in future studies that combine regional volumes, cortical thickness, cortical complexity, and microstructural measures to better characterize the neuroanatomical substrates of APA impairment and FoG.

Although our sample size was comparable to previous FoG neuroimaging studies, it remains modest, particularly for subgroup correlations; therefore, the findings should be interpreted as exploratory brain–behaviour associations rather than definitive regional biomarkers. In addition, all participants initiated gait with the right foot, which standardized the task but prevented us from testing whether associations were influenced by the most affected body side or by lateralized basal ganglia–cortical pathology, an important issue given evidence that right STN and right SMA circuits may contribute to axial and gait control (Di Giulio et al. 2019; Butler et al. 2025; Wróbel et al. 2025). Finally, PD + FoG participants had higher L‐DOPA equivalent doses than PD − FoG participants. This likely reflects greater disease burden and treatment requirement rather than a direct cause of morphometric change, consistent with evidence that levodopa does not modify PD progression (Verschuur et al. 2019); however, medication‐related effects on brain structure cannot be fully excluded.

In conclusion, this study suggests that gait initiation impairment in PD is associated with distributed morphometric alterations rather than a single regional substrate. APA duration was mainly related to striato–thalamo–prefrontal and cingulate volumes, whereas APA mediolateral scaling showed a more specific association with SMA volume in PD + FoG. These findings reinforce the idea that FoG reflects the interaction between motor gating, executive control, and context‐dependent postural preparation. Clinically, APA measures may provide useful biomechanical markers for capturing subtle dysfunction in gait initiation control and identifying neural systems that could be targeted by rehabilitation or neuromodulation strategies. Interventions aimed at improving APA timing and scaling, especially through SMA/fronto‐striatal engagement, external cueing, and cognitive‐motor training, may help optimize step initiation and reduce freezing‐related gait failure in PD.

## Author Contributions

**Andrea Cristina de Lima‐Pardini:** conceptualization, investigation. **Luana dos Santos de Oliveira:** conceptualization, methodology, writing – original draft. **Fabio Augusto Barbieri:** validation, writing – review and editing. **Daniel Boari Coelho:** conceptualization, writing – review and editing, supervision, project administration. **Mariana Penteado Nucci:** conceptualization, methodology, software, formal analysis, writing – review and editing. **Caroline Ribeiro de Souza:** investigation, writing – review and editing. **Carla Silva‐Batista:** investigation, writing – review and editing.

## Funding

This study was supported by the Fundação de Amparo à Pesquisa do Estado de São Paulo (FAPESP, #2024/04859‐0), the Universidade Federal do ABC (UFABC/Brazil), by Coordenação de Aperfeiçoamento de Pessoal de Nível Superior (CAPES/Brazil), and the Conselho Nacional de Desenvolvimento Científico e Tecnológico (CNPq, #306638/2023‐1).

## Conflicts of Interest

The authors declare no conflicts of interest.

## Supporting information




**Data S1:** Supporting information.

## Data Availability

Data will be provided by the corresponding author upon request.
