# Supplementary material for: Brain Morphometry Fingerprints are Associated with Gait Initiation Deficits in Parkinson’s Disease and Freezing of Gait
Source: Eur J Neurosci. 2026 Aug 4;64(3):e70647. doi: 10.1111/ejn.70647 (PMC13437768; doi:10.1111/ejn.70647)
Supplement: Supplementary file 1 [file EJN-64-e70647-s001.docx]

Partial Spearman’s rank correlation coefficients results with UPDRS-III and daily levodopa dose entered as covariates, both across all participants and separately within the PD+FoG and PD−FoG groups. These analyzes tested associations between clinical freezing severity (N-FoGq), APA-derived biomechanical variables (APA duration, APA mediolateral amplitude, and step length), and FreeSurfer-derived regional brain volumes and cortical thickness measures.

All participants

|  |  | Spearman's rho | p | Effect size (Fisher's z) |
| --- | --- | --- | --- | --- |
| APA duration | Caudate volume | -0.328 | 0.059 | -0.34 |
| APA duration | Putamen volume | -0.399* | 0.019 | -0.422 |
| APA duration | Pallidum volume | -0.302 | 0.082 | -0.312 |
| APA duration | Thalamus volume | -0.457** | 0.007 | -0.494 |
| APA duration | Accumbens volume | -0.105 | 0.554 | -0.106 |
| APA duration | Brain-Stem volume | -0.054 | 0.76 | -0.054 |
| APA duration | Amygdala volume | -0.216 | 0.221 | -0.219 |
| APA duration | SMA volume | 0.055 | 0.757 | 0.055 |
| APA duration | DLPFC volume | -0.382* | 0.026 | -0.403 |
| APA duration | Anterior mid-cingulate volume | -0.275 | 0.116 | -0.282 |
| APA duration | Frontopolar volume | -0.302 | 0.082 | -0.312 |
| APA duration | M1 volume | -0.302 | 0.083 | -0.312 |
| APA duration | Insula volume | -0.264 | 0.131 | -0.271 |
| APA duration | Cerebellum White-Matter volume | 0.041 | 0.819 | 0.041 |
| APA duration | Cerebellum Cortex volume | 0.126 | 0.478 | 0.127 |
| APA duration | SMA_thickness | 0.163 | 0.356 | 0.165 |
| APA duration | DLPFC_thickness | -0.092 | 0.606 | -0.092 |
| APA duration | Anterior mid-cingulate_thickness | -0.334 | 0.054 | -0.347 |
| APA duration | Frontopolar_thickness | -0.054 | 0.762 | -0.054 |
| APA duration | M1_thickness | 0.046 | 0.794 | 0.046 |
| APA duration | Insula_thickness | -0.078 | 0.661 | -0.078 |
| APA amplitude | Caudate volume | 0.089 | 0.617 | 0.089 |
| APA amplitude | Putamen volume | -0.068 | 0.704 | -0.068 |
| APA amplitude | Pallidum volume | -0.151 | 0.395 | -0.152 |
| APA amplitude | Thalamus volume | -0.034 | 0.85 | -0.034 |
| APA amplitude | Accumbens volume | -0.263 | 0.134 | -0.269 |
| APA amplitude | Brain-Stem volume | -0.025 | 0.886 | -0.025 |
| APA amplitude | Amygdala volume | -0.195 | 0.268 | -0.198 |
| APA amplitude | SMA volume | 0.205 | 0.246 | 0.208 |
| APA amplitude | DLPFC volume | 0.028 | 0.877 | 0.028 |
| APA amplitude | Anterior mid-cingulate volume | -0.009 | 0.96 | -0.009 |
| APA amplitude | Frontopolar volume | -0.058 | 0.743 | -0.058 |
| APA amplitude | M1 volume | 0.096 | 0.59 | 0.096 |
| APA amplitude | Insula volume | 0.006 | 0.971 | 0.006 |
| APA amplitude | Cerebellum White-Matter volume | 0.008 | 0.962 | 0.008 |
| APA amplitude | Cerebellum Cortex volume | -0.163 | 0.357 | -0.165 |
| APA amplitude | SMA_thickness | -0.071 | 0.69 | -0.071 |
| APA amplitude | DLPFC_thickness | -0.024 | 0.892 | -0.024 |
| APA amplitude | Anterior mid-cingulate_thickness | -0.212 | 0.229 | -0.215 |
| APA amplitude | Frontopolar_thickness | -0.383* | 0.025 | -0.404 |
| APA amplitude | M1_thickness | -0.176 | 0.32 | -0.178 |
| APA amplitude | Insula_thickness | -0.069 | 0.7 | -0.069 |
| Step length | Caudate volume | -0.1 | 0.573 | -0.1 |
| Step length | Putamen volume | 0.04 | 0.824 | 0.04 |
| Step length | Pallidum volume | 0.238 | 0.174 | 0.243 |
| Step length | Thalamus volume | -0.102 | 0.567 | -0.102 |
| Step length | Accumbens volume | -0.131 | 0.459 | -0.132 |
| Step length | Brain-Stem volume | -0.087 | 0.625 | -0.087 |
| Step length | Amygdala volume | 0.025 | 0.887 | 0.025 |
| Step length | SMA volume | 0.18 | 0.307 | 0.182 |
| Step length | DLPFC volume | 0.008 | 0.965 | 0.008 |
| Step length | Anterior mid-cingulate volume | -0.002 | 0.993 | -0.002 |
| Step length | Frontopolar volume | 0.133 | 0.453 | 0.134 |
| Step length | M1 volume | 0.201 | 0.255 | 0.204 |
| Step length | Insula volume | -0.222 | 0.208 | -0.225 |
| Step length | Cerebellum White-Matter volume | 0.152 | 0.391 | 0.153 |
| Step length | Cerebellum Cortex volume | 0.160 | 0.366 | 0.161 |
| Step length | SMA_thickness | -0.118 | 0.507 | -0.118 |
| Step length | DLPFC_thickness | -0.227 | 0.197 | -0.231 |
| Step length | Anterior mid-cingulate_thickness | -0.143 | 0.419 | -0.144 |
| Step length | Frontopolar_thickness | -0.144 | 0.418 | -0.145 |
| Step length | M1_thickness | -0.128 | 0.47 | -0.129 |
| Step length | Insula_thickness | -0.046 | 0.798 | -0.046 |
|  | | | | |

PD+FoG

|  |  | Spearman's rho | p | Effect size (Fisher's z) |
| --- | --- | --- | --- | --- |
| APA duration | Caudate volume | -0.417 | 0.096 | -0.444 |
| APA duration | Putamen volume | -0.378 | 0.135 | -0.397 |
| APA duration | Pallidum volume | -0.377 | 0.136 | -0.397 |
| APA duration | Thalamus volume | -0.662** | 0.004 | -0.796 |
| APA duration | Accumbens volume | -0.14 | 0.591 | -0.141 |
| APA duration | Brain-Stem volume | -0.09 | 0.731 | -0.09 |
| APA duration | Amygdala volume | -0.113 | 0.665 | -0.114 |
| APA duration | SMA volume | 0.125 | 0.633 | 0.126 |
| APA duration | DLPFC volume | -0.509* | 0.037 | -0.562 |
| APA duration | Anterior mid-cingulate volume | -0.558* | 0.02 | -0.63 |
| APA duration | Frontopolar volume | -0.085 | 0.747 | -0.085 |
| APA duration | M1 volume | -0.183 | 0.483 | -0.185 |
| APA duration | Insula volume | -0.278 | 0.28 | -0.286 |
| APA duration | Cerebellum White-Matter volume | -0.034 | 0.897 | -0.034 |
| APA duration | Cerebellum Cortex volume | 0.172 | 0.509 | 0.174 |
| APA duration | SMA_thickness | 0.354 | 0.163 | 0.37 |
| APA duration | DLPFC_thickness | 0.131 | 0.615 | 0.132 |
| APA duration | Anterior mid-cingulate_thickness | -0.241 | 0.352 | -0.246 |
| APA duration | Frontopolar_thickness | 0.056 | 0.83 | 0.056 |
| APA duration | M1_thickness | 0.331 | 0.195 | 0.344 |
| APA duration | Insula_thickness | -0.245 | 0.344 | -0.25 |
| APA amplitude | Caudate volume | 0.074 | 0.777 | 0.074 |
| APA amplitude | Putamen volume | -0.202 | 0.437 | -0.205 |
| APA amplitude | Pallidum volume | -0.433 | 0.082 | -0.464 |
| APA amplitude | Thalamus volume | 0.292 | 0.256 | 0.3 |
| APA amplitude | Accumbens volume | -0.236 | 0.362 | -0.24 |
| APA amplitude | Brain-Stem volume | 0.148 | 0.57 | 0.149 |
| APA amplitude | Amygdala volume | -0.1 | 0.702 | -0.1 |
| APA amplitude | SMA volume | 0.504* | 0.039 | 0.555 |
| APA amplitude | DLPFC volume | 0.335 | 0.189 | 0.349 |
| APA amplitude | Anterior mid-cingulate volume | 0.225 | 0.386 | 0.229 |
| APA amplitude | Frontopolar volume | 0.012 | 0.964 | 0.012 |
| APA amplitude | M1 volume | 0.18 | 0.49 | 0.182 |
| APA amplitude | Insula volume | 0.136 | 0.602 | 0.137 |
| APA amplitude | Cerebellum White-Matter volume | -0.326 | 0.202 | -0.338 |
| APA amplitude | Cerebellum Cortex volume | -0.369 | 0.145 | -0.387 |
| APA amplitude | SMA_thickness | 0.225 | 0.386 | 0.229 |
| APA amplitude | DLPFC_thickness | 0.04 | 0.88 | 0.04 |
| APA amplitude | Anterior mid-cingulate_thickness | -0.148 | 0.57 | -0.15 |
| APA amplitude | Frontopolar_thickness | -0.307 | 0.231 | -0.317 |
| APA amplitude | M1_thickness | 0.021 | 0.938 | 0.021 |
| APA amplitude | Insula_thickness | 0.108 | 0.68 | 0.108 |
| Step length | Caudate volume | 0.037 | 0.889 | 0.037 |
| Step length | Putamen volume | 0.182 | 0.484 | 0.184 |
| Step length | Pallidum volume | 0.235 | 0.364 | 0.239 |
| Step length | Thalamus volume | -0.052 | 0.843 | -0.052 |
| Step length | Accumbens volume | -0.102 | 0.698 | -0.102 |
| Step length | Brain-Stem volume | -0.109 | 0.677 | -0.109 |
| Step length | Amygdala volume | 0.006 | 0.981 | 0.006 |
| Step length | SMA volume | -0.017 | 0.948 | -0.017 |
| Step length | DLPFC volume | -0.047 | 0.857 | -0.047 |
| Step length | Anterior mid-cingulate volume | -0.284 | 0.269 | -0.292 |
| Step length | Frontopolar volume | 0.048 | 0.855 | 0.048 |
| Step length | M1 volume | -0.048 | 0.854 | -0.048 |
| Step length | Insula volume | -0.502* | 0.04 | -0.552 |
| Step length | Cerebellum White-Matter volume | -0.220 | 0.396 | -0.224 |
| Step length | Cerebellum Cortex volume | 0.197 | 0.448 | 0.200 |
| Step length | SMA_thickness | -0.381 | 0.131 | -0.402 |
| Step length | DLPFC_thickness | -0.347 | 0.172 | -0.362 |
| Step length | Anterior mid-cingulate_thickness | -0.103 | 0.694 | -0.103 |
| Step length | Frontopolar_thickness | -0.263 | 0.308 | -0.269 |
| Step length | M1_thickness | -0.108 | 0.679 | -0.109 |
| Step length | Insula_thickness | -0.023 | 0.931 | -0.023 |
|  | | | | |

PD-FoG

|  |  | Spearman's rho | p | Effect size (Fisher's z) |
| --- | --- | --- | --- | --- |
| APA duration | Caudate volume | -0.201 | 0.472 | -0.204 |
| APA duration | Putamen volume | -0.444 | 0.098 | -0.477 |
| APA duration | Pallidum volume | -0.28 | 0.312 | -0.288 |
| APA duration | Thalamus volume | -0.337 | 0.219 | -0.351 |
| APA duration | Accumbens volume | -0.136 | 0.63 | -0.137 |
| APA duration | Brain-Stem volume | -0.258 | 0.354 | -0.264 |
| APA duration | Amygdala volume | -0.509 | 0.053 | -0.562 |
| APA duration | SMA volume | -0.052 | 0.854 | -0.052 |
| APA duration | DLPFC volume | -0.45 | 0.093 | -0.484 |
| APA duration | Anterior mid-cingulate volume | -0.118 | 0.674 | -0.119 |
| APA duration | Frontopolar volume | -0.689** | 0.004 | -0.847 |
| APA duration | M1 volume | -0.422 | 0.117 | -0.45 |
| APA duration | Insula volume | -0.425 | 0.114 | -0.454 |
| APA duration | Cerebellum White-Matter volume | 0.037 | 0.895 | 0.037 |
| APA duration | Cerebellum Cortex volume | -0.073 | 0.795 | -0.073 |
| APA duration | SMA_thickness | -0.045 | 0.874 | -0.045 |
| APA duration | DLPFC_thickness | -0.471 | 0.077 | -0.511 |
| APA duration | Anterior mid-cingulate_thickness | -0.572* | 0.026 | -0.651 |
| APA duration | Frontopolar_thickness | -0.408 | 0.132 | -0.433 |
| APA duration | M1_thickness | -0.225 | 0.42 | -0.229 |
| APA duration | Insula_thickness | -0.049 | 0.861 | -0.049 |
| APA amplitude | Caudate volume | 0.057 | 0.84 | 0.057 |
| APA amplitude | Putamen volume | 0.158 | 0.574 | 0.159 |
| APA amplitude | Pallidum volume | 0.25 | 0.369 | 0.255 |
| APA amplitude | Thalamus volume | -0.011 | 0.968 | -0.011 |
| APA amplitude | Accumbens volume | -0.147 | 0.601 | -0.148 |
| APA amplitude | Brain-Stem volume | 0.138 | 0.623 | 0.139 |
| APA amplitude | Amygdala volume | 0.041 | 0.885 | 0.041 |
| APA amplitude | SMA volume | -0.132 | 0.639 | -0.133 |
| APA amplitude | DLPFC volume | -0.18 | 0.522 | -0.182 |
| APA amplitude | Anterior mid-cingulate volume | 0.033 | 0.908 | 0.033 |
| APA amplitude | Frontopolar volume | 0.212 | 0.448 | 0.215 |
| APA amplitude | M1 volume | 0.034 | 0.905 | 0.034 |
| APA amplitude | Insula volume | 0.005 | 0.986 | 0.005 |
| APA amplitude | Cerebellum White-Matter volume | 0.239 | 0.390 | 0.244 |
| APA amplitude | Cerebellum Cortex volume | -0.131 | 0.642 | -0.132 |
| APA amplitude | SMA_thickness | -0.222 | 0.426 | -0.226 |
| APA amplitude | DLPFC_thickness | 0.021 | 0.941 | 0.021 |
| APA amplitude | Anterior mid-cingulate_thickness | -0.103 | 0.715 | -0.104 |
| APA amplitude | Frontopolar_thickness | -0.308 | 0.263 | -0.319 |
| APA amplitude | M1_thickness | -0.15 | 0.593 | -0.151 |
| APA amplitude | Insula_thickness | -0.253 | 0.363 | -0.258 |
| Step length | Caudate volume | -0.287 | 0.299 | -0.296 |
| Step length | Putamen volume | -0.006 | 0.983 | -0.006 |
| Step length | Pallidum volume | 0.26 | 0.349 | 0.266 |
| Step length | Thalamus volume | 0.011 | 0.969 | 0.011 |
| Step length | Accumbens volume | 0.076 | 0.788 | 0.076 |
| Step length | Brain-Stem volume | 0.348 | 0.203 | 0.364 |
| Step length | Amygdala volume | 0.526* | 0.044 | 0.584 |
| Step length | SMA volume | 0.257 | 0.356 | 0.262 |
| Step length | DLPFC volume | 0.347 | 0.205 | 0.362 |
| Step length | Anterior mid-cingulate volume | 0.468 | 0.079 | 0.507 |
| Step length | Frontopolar volume | 0.322 | 0.243 | 0.333 |
| Step length | M1 volume | 0.476 | 0.073 | 0.518 |
| Step length | Insula volume | 0.166 | 0.553 | 0.168 |
| Step length | SMA_thickness | 0.184 | 0.513 | 0.186 |
| Step length | DLPFC_thickness | -0.026 | 0.926 | -0.026 |
| Step length | Anterior mid-cingulate_thickness | -0.109 | 0.7 | -0.109 |
| Step length | Frontopolar_thickness | 0.178 | 0.527 | 0.179 |
| Step length | M1_thickness | 0.034 | 0.903 | 0.034 |
| Step length | Insula_thickness | 0.25 | 0.368 | 0.256 |
|  | | | | |
